# Meta-Analysis: The Efficacy and Safety of Paricalcitol for the Treatment of Secondary Hyperparathyroidism and Proteinuria in Chronic Kidney Disease

**DOI:** 10.1155/2013/320560

**Published:** 2012-12-27

**Authors:** Tianzhao Han, Gong Rong, Dayong Quan, Ying Shu, Zhu Liang, Ninglan She, Manli Liu, Bing Yang, Gong Cheng, Yongman Lv, Leonard Stern

**Affiliations:** ^1^The Department of Nephrology, The Third People's Hospital of Chengdu, The Chongqing Medical University, Chengdu 610031, China; ^2^Department of Nephrology, Tongji Hospital, Tongji Medical College, Huazhong University of Science and Technology, Wuhan 430000, China; ^3^Division of Nephrology, Department of Medicine, Columbia University Medical Center, 622 West 168th Street, PH 4-124, New York, NY 10032, USA

## Abstract

*Introduction*. Previous studies have demonstrated the safety and efficacy of using Paricalcitol for the treatment of secondary hyperparathyroidism (SHPT) in patients on dialysis. The aim of the current meta-analysis was to assess the safety and efficacy of Paricalcitol for the management of SHPT in patients with chronic kidney disease (CKD) not yet on dialysis. A secondary aim was to determine if sufficient data was available to assess the effect of Paricalcitol for the management of proteinuria. *Methods*. A meta-analysis was conducted using the Cochrane Collaboration's RevMan 4.2 software. *Results*. Paricalcitol is effective in lowering PTH in patients with CKD not yet on dialysis and is also effective in lowering proteinuria in diabetic CKD patients. However, we uncovered a safety signal identifying an elevated calcium phosphate product and a trend towards the development of hypercalcemia. A phosphate elevation was not demonstrated because the target used in the clinical studies was a *P* > 5.5 mg/dl, a value appropriate for dialysis patients and not CKD patients. *Conclusion*. Although Paricalcitol is effective in lowering PTH, we advise caution in the use of any active Vitamin D analogues in patients with CKD because of the potential risk of exacerbating vascular calcification.

## 1. Introduction

One of the greatest therapeutic challenges in the chronic kidney disease population is the management of bone and mineral metabolic parameters in order to preserve bone integrity, minimize cardiovascular calcification, and manage serum levels of parathyroid hormone (PTH), calcium and phosphorus. The cornerstone of this condition is characterized by the CKD-MBD (chronic kidney disease-mineral bone disorder) syndrome where there is secondary hyperparathyroidism (SHPT), manifested by parathyroid hyperplasia and upregulated synthesis and secretion of PTH [1, 2]. In addition, there is an elevation of the serum phosphate, a reduction in serum calcium, and an absolute reduction of active vitamin D (calcitriol) levels caused by a reduction of the synthetic 1a-hydroxylase encoded by the CYP27B1 gene and an increase in the catabolic 24a-hydroxylase encoded by the CYP 24 gene. Both of these enzymatic changes are characteristically present in CKD and are very likely mediated by the high levels of FGF23 also characteristically present in CKD [3]. The other features of this syndrome include renal osteodystrophy where an abnormality of bone anabolism causes high bone turnover disease, fractures, vascular calcification, and cardiovascular complications. Slowing the rate of progression towards end-stage renal disease is one of the key goals of medical intervention in this patient group.

Secondary hyperparathyroidism (SHPT) is a common and early complication of CKD. Targeting SHPT in patients with CKD and end stage renal disease on dialysis with active vitamin D analogues such as Paricalcitol has been the subject of multiple research studies in patients. Numerous studies of mixed quality, targeting various surrogate outcome measures have been published clearly demonstrating the biologic importance of the therapy [4–7, 10]. Active vitamin D analogues including Paricalcitol have shown demonstrably favorable effects on SHPT [4, 6–11] and proteinuria [4–6, 12–14]. In these studies, no clinically important or statistically evident change in eGFR has been shown [4–6, 8, 9, 12, 14]. The active vitamin D analogues have also shown clear evidence for decreases in cardiovascular events [15], and improved survival in hemodialysis patients. [16, 17]. The majority of the published research data, however, has been obtained in patients on dialysis [7, 10, 11, 15–17]. The role of treatment with Paricalcitol in CKD targeting early SHPT is less clear [4–6, 8, 9]. A previously published meta-analysis summarized the efficacy of Paricalcitol therapy for chronic kidney disease combining the data for patients receiving and not yet on dialysis and concluded that Paricalcitol suppresses iPTH and lowers proteinuria in patients with stages 2–5 CKD without an increased risk of adverse events [14]. At least one study has presented evidence for the use of vitamin D analogues in the prevention of vascular calcification [18]. 

One of the many biologic actions of active vitamin D is to cause an increase in the amount of intestinal calcium and phosphorus absorption, resulting in hypercalcemia and hyperphosphatemia. At higher than physiologic dosages, active vitamin D may actually increase bone resorption. Paricalcitol, a synthetic vitamin D analogue engineered to effectively suppress secretion of PTH with fewer hypercalcemic and hyperphosphatemic side effects, has been shown to effectively reduce PTH and also reduce proteinuria in recent studies in patients with CKD [4–6, 12, 14, 16, 17]. The goal of the present meta-analysis was to evaluate the efficacy and safety of treatment with Paricalcitol in the management of SHPT, proteinuria, and preservation of renal function in patients with CKD. In particular, we wanted to evaluate whether there is sufficient published data to recommend treatment with Paricalcitol to patients with CKD and SHPT not yet on dialysis.

## 2. Methods

### 2.1. Data Sources and Searches

The literature searches for randomized, controlled trials (RCTs) of Paricalcitol in CKD were retrieved from PubMed, Medline, EMBASE, Elsevier Science, Karger, Free Medical Journals, BMJ, Nature and CNKI between 1993 and 2009 by using the search strategy “Paricalcitol Limits Activated: Humans, Randomized Controlled Trial.” The Reference sections of included articles were reviewed for other potentially relevant citations. Finally, the authors of included studies were personally contacted to obtain further information.

### 2.2. Study Selection

#### 2.2.1. Inclusion Criteria

Only randomized, controlled trials were considered for inclusion in this analysis. Other criteria included the following. (1) Treatment group received Paricalcitol and the control group received placebo. (2) Definitions of proteinuria, hypercalcemia and hyperphosphatemia were similar in all reports. (3) Each study had the inclusion/exclusion criteria and participants were considered eligible. The authors must have given the size of their samples, a significance level, and their 95% confidence intervals (CIs). The methods of analysis using analysis of covariance or Fisher's exact test were statistically acceptable. (4) Studies of other vitamin D compounds or in other non-CKD disease states were excluded.

#### 2.2.2. Exclusion Criteria

Patients were excluded if they failed to meet the inclusion criteria or if they failed to complete the study protocol. We also excluded animal studies.

#### 2.2.3. Efficacy Indices

*Change in iPTH.* Defined as achieving a greater than or equal to 30% decrease in iPTH from baseline for two consecutive measures. 

*Proteinuria*. Defined as a statistically significant decrease in urinary protein-creatinine ratio or urinary albumin-creatinine ratio.

Δ*eGFR*. The mean change in eGFR from baseline to final visit. 

*Hypercalcemia*. Defined as two consecutive calcium measurements of greater than 2.62 mmol/L or 10.5 mg/dL.

*Hyperphosphatemia*. Defined as two consecutive phosphorus measurements of greater than 5.5 mg/dL.

*Elevation in Calcium × Phosphorus Product*. Defined as two consecutive calcium × phosphate product values of greater than 55 mg^2^/dL^2^.

### 2.3. Data Extraction and Quality Assessment

Three independent authors extracted relevant data from eligible studies. Discrepancies were resolved by discussion and by referencing the original report. Two independent authors assessed each trial using the Jadad rating scale [19] and referred to the Cochrane Reviewers' Handbook 4.2.6 about the quality of randomized controlled trials (randomization, blinding, withdrawal and loss, allocation concealment, and intentional Analysis—A: adequate, B: unclear, C: inadequate, and D: not used) [20].

### 2.4. Data Synthesis and Analysis

A meta-analysis was conducted using the Cochrane Collaboration's RevMan 4.2 software. A test of heterogeneity was assessed by the chi-square test (*P* value and *I* ^2^), which describes the percentage of variability in the effect and estimates the contribution of heterogeneity rather than by chance [21, 22]. We summarized treatment effects as relative risks (RRs) for categorical variables and weighted mean differences for continuous variables, with 95% CIs. If no heterogeneity existed among studies (*P* ≥ 0.05 and *I* ^2^ > 0.5), the fixed effect model was used. An *I* ^2^ = 0, indicated that the variation was caused by sampling error; A *I* ^2^ < 0.25, indicated a slight degree of heterogeneity; A *I* ^2^ > 0.25– <0.5, indicated a moderate degree of heterogeneity; A *I* ^2^ > 0.5, indicated a high degree of heterogeneity [23]. If *I* ^2^ > 0.5 or *P* < 0.05, the heterogeneity among these studies was considered statistically significant and a descriptive analysis was employed. 

## 3. Results

A total of 25 articles were retrieved in the initial search. We found 1 ongoing study (VITAL study) [23] and fortunately obtained results about the published VITAL study from the corresponding author [12]. Examination of the abstracts and full texts allowed us to exclude non case-control studies or studies where the participants did not have CKD, leaving 9 articles that form the basis of this meta-analysis.

### 3.1. Trial Characteristics

The 9 studies included a total of 1113 participants; 20 participants did not complete the protocol and are excluded leaving 1093 participants included in this meta-analysis. 58.2% had diabetic kidney disease, 20.6% had nondiabetic kidney disease, and the remainders were not characterized. The characteristics of the nine studies and the efficacy parameters are summarized in Tables 1(a) and 1(b), respectively. 

### 3.2. Trial Quality

We assessed the quality of included studies using the Jadad rating scale [19] and referred to the Cochrane Reviewer's Handbook 4.2.6 for guidelines used to rate the quality of randomized controlled trials [20] (Table 2). The main factors influencing quality were allocation concealment, intentional analysis, withdrawal, and dropout. The primary reasons described for premature withdrawal of these patients were kidney transplantation, increase in iPTH levels, unblinding, and failure to complete a scheduled protocol visit. Each study received a grade of A or B. The Jadad rating score was assigned from 2 to 5 points. 

### 3.3. Meta-Analysis Results

#### 3.3.1. Two Consecutive Decreases of Greater Than or Equal to 30% in iPTH

**Figure 1:**
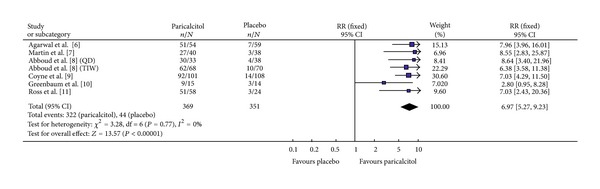
Comparison of the probability of achieving ≥30% decrease in iPTH from baseline for two consecutive measures.

The six studies that compared this efficacy index included a total of 720 participants; 369 and 351 treated with Paricalcitol and placebo, respectively (Figure 1). All six studies had homogeneity (heterozygosity test, *χ* ^2^ = 3.28, *P* = 0.77, *I* ^2^ = 0%). When the fixed-effect model was used to merge RR values, the pooled RR was 6.97 (95% CI 5.27–9.23, *Z* = 13.57, *P* < 0.00001; Table 3). This indicated that the Paricalcitol treated patients had a statistically significant sustained reduction in serum iPTH levels during the observation period.

#### 3.3.2. ΔeGFR

**Figure 2:**
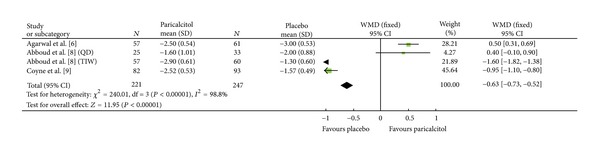
Comparison of the mean change in eGFR (mL/min/1.73 m^2^) from baseline to final visit.

Among the three studies that reported this efficacy index, a total of 468 patients were included; 221 and 247 in the Paricalcitol and placebo groups, respectively (Figure 2). All three studies had heterogeneity (heterozygosity test, *χ* ^2^ = 240.01, *P* < 0.00001, *I* ^2^ = 98.8%; Table 3). Because of this high heterogeneity, the effect of Paricalcitol on the ΔeGFR using formal meta-analysis technique is not certain. Using a more conventional descriptive analysis, the data from each study (Table 4) show no statistically significant difference between the Paricalcitol-treated and placebo groups implying that Paricalcitol had no negative impact on renal function.

#### 3.3.3. Proteinuria

**Figure 3:**
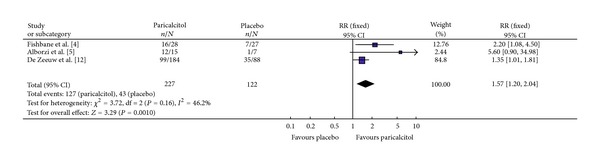
Comparison of the reduction in proteinuria.

Three studies included this efficacy index with a total of 349 participants; 227 and 122 in the Paricalcitol and placebo groups, respectively (Figure 3). The majority (88.6%) had diabetic kidney disease. All three studies had homogeneity (heterozygosity test, *χ* ^2^ = 3.72, *P* = 0.16, *I* ^2^ = 46.2%). When the fixed-effect model was used to merge RR values, the pooled RR was 1.57 (95% CI 1.20–2.04, *Z* = 3.29, *P* = 0.0010; Table 3). This indicated that Paricalcitol-treated patients with diabetic CKD had a statistically significant reduction in proteinuria compared to placebo.

**Figure 4:**
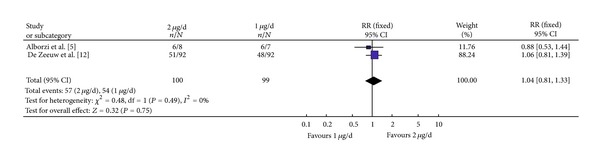
Comparison of the reduction in proteinuria netween the 1 ug/d and 2 ug/d groups.

Two studies, with 199 participants reported the effect of Paricalcitol with varying dosages. 99 patients received 1 microgram and 100 received a 2 microgram dose (Figure 4). Both studies had homogeneity (heterozygosity test, *χ* ^2^ = 0.48, *P* = 0.49, *I* ^2^ = 0%). When the fixed-effect model was used to merge RR values, the pooled RR was 1.04 (95% CI 0.81–1.33, *Z* = 0.32, *P* = 0.75; Figure 4). Comparing the 1 and 2 microgram Paricalcitol-treated groups; there was no statistically significant difference in proteinuria reduction. 

#### 3.3.4. Hypercalcemia

**Figure 5:**
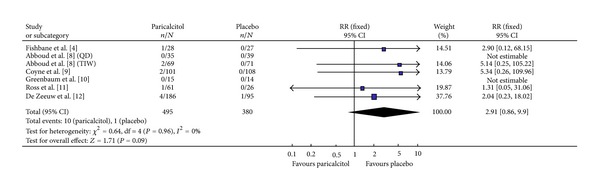
Incidence of hypercalcemia.

Among the six studies where this efficacy parameter is reported, 875 participants were evaluated for the incidence of hypercalcemia; 495 and 380 in Paricalcitol and placebo groups, respectively (Figure 5). All six studies had homogeneity (heterozygosity test, *χ* ^2^ = 0.64, *P* = 0.96, *I* ^2^ = 0%). When the fixed-effect model was used to merge RR values, the pooled RR was 2.91 (95% CI 0.86–9.90, *Z* = 1.71, *P* = 0.09; Table 3). This indicated that there was no statistically significant difference in the incidence of hypercalcemia between the Paricalcitol and placebo groups though a trend towards hypercalcemia was evident in the Paricalcitol-treated groups, where 10 of 495 in the Paricalcitol group and 1 of 380 in the placebo group developed hypercalcemia. There was insufficient data to determine a dose-response effect comparing 1 ug versus 2 ug dosing.

#### 3.3.5. Hyperphosphatemia

**Figure 6:**
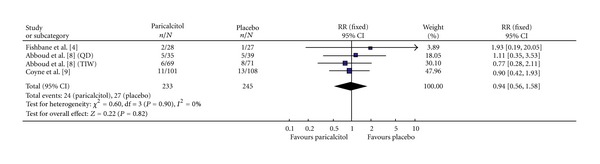
Incidence of hyperphosphatemia.

Among the three studies reporting this efficacy parameter, 478 participants were evaluated for the incidence of hyperphosphatemia; 233 and 245 in the Paricalcitol and placebo groups, respectively (Figure 6). All three studies had homogeneity (heterozygosity test, *χ* ^2^ = 0.60, *P* = 0.90, *I* ^2^ = 0%). When the fixed-effect model was used to merge RR values, the pooled RR was 0.94 (95% CI 0.56–1.58, *Z* = 0.22, *P* = 0.82; Table 3). This indicated that there was no statistically significant difference in the incidence of hyperphosphatemia between the Paricalcitol and placebo groups. The studies included, used 5.5 mg/dL, as the upper limit of the acceptable range for the serum phosphate. The current KDIGO guidelines recommend targeting the serum phosphate to the normal range, in this case a level <4.0 mg/dL [24].

#### 3.3.6. Elevation in Calcium × Phosphorus (Ca × P) Product

**Figure 7:**
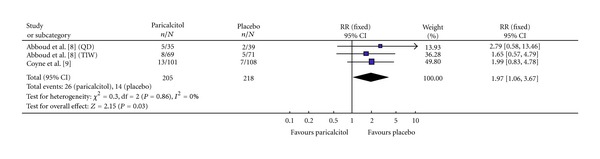
Incidence of an elevation in calcium × phosphorus product.

In the two studies reporting this efficacy parameter, 423 participants were evaluated for a change in calcium × phosphorus product levels, 205 and 218 in the Paricalcitol and placebo groups, respectively (Figure 7). Both studies had homogeneity (heterozygosity test, *χ* ^2^ = 0.30, *P* = 0.86, *I* ^2^ = 0%). When the fixed-effect model was used to merge RR values, the pooled RR was 1.97 (95% CI 1.06–3.67, *Z* = 2.15, *P* = 0.03; Table 3). While, the data shows that there was no statistically significant difference in the incidence of an elevation in Ca × P product between the Paricalcitol and placebo groups for each individual study (Table 5), the pooled data in the meta-analysis do show a statistically significant increase in the incidence of an elevated Ca × P product between the Paricalcitol- and placebo-treated groups (*P* = 0.03).

## 4. Discussion

The CKD-MBD syndrome characteristic of chronic kidney disease (CKD) of virtually any etiology imposes the burden of excess mineral retention enhancing cardiovascular risk by promoting the development of vascular calcification [13, 25–27]. The hallmark biochemical abnormalities identified in CKD are a reduced level of active vitamin D which results in an elevated blood level of PTH by upregulation of the synthesis and secretion of parathyroid hormone [27]. Targeting PTH synthesis by treating the active vitamin D insufficiency is the generally accepted standard of care [28, 29]. However, the treatment with active vitamin D analogues may promote further retention of calcium and phosphate and potentially worsen the cardiovascular risk profile of the patients with CKD being treated. Paricalcitol is a synthetic active vitamin D analog chemically designed to limit the absorption of calcium and phosphate by the intestine [30]. In low doses, Paricalcitol results in a 10-fold reduction of calcium absorption compared to calcitriol [31]. Paricalcitol acts as an active agonist for the vitamin D receptor and in the parathyroid gland negatively regulates the gene transcription for PTH thus lowering the blood parathyroid hormone level [32–35]. However, Paricalcitol like all currently available active vitamin D analogues directly binds to the VDR in many tissues [36]. Intestinal activation of the VDR can cause hypercalcemia and hyperphosphatemia by enhanced intestinal absorption. Hyperphosphatemia by itself has been associated with increased mortality in patients on dialysis as well as those with CKD not yet on dialysis [36, 37]. Paricalcitol has more selective activation of VDR in the parathyroid gland over that in the intestine and bone and offers the possibility of minimizing the risk of hypercalcemia and hyperphosphatemia, while still significantly reducing PTH [30, 38, 39].

The goal of the current meta-analysis was to assess whether there is sufficient evidence-based data to recommend Paricalcitol for the management of SHPT in chronic kidney disease patients not yet on dialysis. A second goal of this meta-analysis was to assess whether there is sufficient evidence-based data to recommend Paricalcitol in the management of proteinuric renal disease. 

The results of the current meta-analysis indicate that Paricalcitol can decrease iPTH levels significantly with a relatively low incidence of hypercalcemia and hyperphosphatemia. However, we noted that there was a statistically significant difference in elevated Ca × P product levels, which was not found in previous studies [8, 9, 14]. A recently published meta-analysis including patients with CKD and patients with ESRD on dialysis did not identify any safety issues [14]. In contrast, our study looked at patients with CKD only and this safety issue was readily identified. Although there is no statistically significant difference in the incidences of hypercalcemia and hyperphosphatemia between Paricalcitol compared to placebo, an element of caution is needed in interpreting this data. Active vitamin D analogues, including Paricalcitol, are very likely to have a dose-dependent effect, with higher doses possibly resulting in both hypercalcemia and hyperphosphatemia potentially contributing to an increase in cardiovascular complications. In the studies included in this review, there was insufficient power to identify a dose-response effect. Furthermore, the studies included used 5.5 mg/dL as the upper limit of the acceptable range for the serum phosphate. This level is recommended in the KDOQI guidelines for patients on dialysis [40]. However, patients with chronic kidney disease usually do not reach such high levels and the current KDIGO guidelines recommend targeting the serum phosphate to the normal range, in this case a level (<4.0 mg/dL) [24]. Thus, the currently available data may grossly underestimate the effect of Paricalcitol on the serum phosphate in this patient group with CKD not on dialysis. 

Paricalcitol has recently been shown to reduce proteinuria in patients with diabetic kidney disease [4–6, 12, 14]. De Zeeuw et al. [12] reported changes in urinary albumin-to-creatinine ratio (UACR) and did not note a dose-response relation with Paricalcitol. Due to the limited number of studies included in this meta-analysis about this component, we also did not observe a significant difference between the 1 microgram Paricalcitol treated groups compared with the 2 microgram Paricalcitol treated groups in reducing proteinuria. The current meta-analysis included three studies [4, 5, 12] that found a significant decrease in proteinuria with Paricalcitol therapy compared with placebo. Agarwal et al. [6] analyzed data from a randomized controlled trial comparing Paricalcitol with placebo for the treatment of secondary hyperparathyroidism in chronic kidney disease. Urinalysis dipstick proteinuria (qualitative) was assessed, and a decrease in proteinuria occurred in 51% of Paricalcitol-treated patients compared with 25% of controls. Szeto et al. [13] studied 10 patients with immunoglobulin. A nephropathy-treated with calcitriol, 0.5 ug, twice-week for 12 weeks and found a significant decrease in proteinuria. Moreover, in studies [4–6, 12, 13], ACE inhibitors and/or ARBs were used in the majority of patients, and the interaction with a decrease in proteinuria was not significant in the patients receiving ACE inhibitors or ARBs. There may be several possible mechanisms of action for this effect on reducing proteinuria, though none are definitive. Results of experimental studies suggest that the reduction in proteinuria induced by Paricalcitol is caused by inhibition of T-cell proliferation and activation [24, 41], reduced cytokine and transforming growth factor *β* production, [42] protection of podocytes [43], and suppression of the renin-angiotensin system [44, 45].

The result of changes in eGFR in the current meta-analysis should also be interpreted with caution, because relevant data for this meta-analysis were highly heterogeneous. The studies selected contained descriptive analyses that supported the hypothesis that there was no clinically or statistically significant difference in eGFR between Paricalcitol and placebo groups. This same observation in reference to eGFR was noted in other studies as well [12–14, 46].

One of the major limitations of this meta-analysis is the inclusion of only a limited number of studies that met the predetermined set of entry criteria. To minimize bias, we thoroughly carried out searches across different databases using explicit criteria for study selection, data analysis, and data abstraction. Not all of the studies used intention to treat analysis, and allocation concealment was adequate in only five studies. The absence of both of these components could potentially lead to bias. In addition, we could not assess a funnel plot to reveal possible publication bias. Furthermore, the current KDIGO guideline for the target serum phosphorus is a normal level usually defined as <4.0 mg/dL [40]. However, each of the studies included in this meta-analysis used a definition of an elevated serum phosphorus as >5.5 mg/dL. The data to recalculate the effect of Paricalcitol using the currently accepted lower serum phosphorus target was not available to us. Lastly, the small number of studies that addressed the use of Paricalcitol for the treatment of proteinuria in patients without diabetic kidney disease contain insufficient data to conclude that Paricalcitol can decrease proteinuria in these conditions.

In conclusion, there is sufficient evidence based data to conclude that Paricalcitol can effectively decrease iPTH levels in patients with CKD not yet on dialysis. We feel that the data evaluating the effects on serum calcium and phosphate are troublesome and our inability to demonstrate adverse events is limited by insufficient power in the analysis. Moreover, the definition of hyperphosphatemia masked the effect on the serum phosphorus rendering this data largely uninterpretable. The data evaluating the effect on the serum calcium although not statistically significant with a *P* < 0.09, definitely showed a trend towards hypercalcemia. This trend was also identified in a previously published meta-analysis which included both patients with CKD and ESRD on dialysis [14]. The statistically significant abnormality highlighting the elevation in Ca × P product supports the notion that clinically significant abnormalities in calcium and phosphate levels may be present with active vitamin D analogue treatment including Paricalcitol. We recommend caution in the use of vitamin D analogues including Paricalcitol in the management of SHPT in CKD patients not on dialysis and advise using the lowest effective dose with careful monitoring for the development of hypercalcemia and hyperphosphatemia.

There is also evidence-based data to conclude that Paricalcitol can lead to a significant reduction in proteinuria in patients with diabetic CKD with no apparent measurable impact on kidney function, but not in the case of patients with nondiabetic kidney disease where there is insufficient data. Due to inherent limitations of meta-analysis, larger association studies or multicentric case-control studies are needed to confirm these findings, especially the effect on the serum phosphate using the currently accepted lower target blood level and the effect on the serum calcium using a larger database with sufficient power analysis.****

## Figures and Tables

**Table:** (a)

Reference	Total number (Paricalcitol/placebo)	Etiology of CKD					
		DM	no DM	Treatment	Dosing regimen	Route of administration	Age
[4]	55 (28/27)	26	29	6-month	1 ug/d and 2 ug/d	Oral	18–85
[5]	24 (16/8)	13	11	1-month	1 ug/d and 2 ug/d	Oral	>18
[6]	118 (57/61)	79	4	24-week	1 ug/d and 2 ug/d or 2 ug TIW and 4 ug TIW	Oral	≥18
[7]	78 (40/38)	—	—	12-week	0.04 ug/kg TIW	Intravenous	22–90
[8] TIW	145 (72/73)	88	57	24-week	2 ug TIW and 4 ug TIW	Oral	≥18
[8] QD	75 (35/40)	41	34	24-week	1 ug/d and 2 ug/d	Oral	≥18
[9]	220 (107/113)	129	91	24-week	1 ug/d and 2 ug/d or 2 ug TIW and 4 ug TIW	Oral	≥18
[10]	29 (15/14)	—	—	12-week	0.04 ug/kg or 0.08 ug/kg TIW	Intravenous	2–20
[11]	88 (61/27)	—	—	12-week	iPTH/60 TIW	Oral	≥18
[12]	281 (186/95)	272	0	24-week	1 ug/d and 2 ug/d	Oral	>20

Reference [8] had different methods of administration and gave us the information, respectively.

TIW: treatment with Paricalcitol or placebo thrice weekly.

QD: treatment with Paricalcitol or placebo once a day.

**Table:** (b)

Reference	[4]	[5]	[6]	[7]	[8] (TIW)	[8] (QD)	[9]	[10]	[11]	[12]	Total number (P/p)
Total number of patients (P/p)	55 (28/27)	24 (16/8)	118 (57/61)	78 (40/38)	145 (72/73)	75 (35/40)	220 (107/113)	29 (15/14)	88 (61/27)	281 (186/95)	1113 (617/496)
30% decrease in iPTH levels for two consecutive measures (P/p)	—	—	113 (54/59)	78 (40/38)	138 (68/70)	71 (33/38)	209 (101/108)	29 (15/14)	82 (58/24)	—	720 (369/351)
Mean eGFR change from baseline to the final visit (P/p)	—	—	118 (57/61)	—	117 (57/60)	58 (25/33)	175 (82/93)	—	—	—	468 (221/247)
Incidence of hypercalcemia (P/p)	55 (28/27)	—	—	—	140 (69/71)	74 (35/39)	209 (101/108)	29 (15/14)	87 (61/26)	281 (186/95)	875 (495/380)
Incidence of hyperphosphatemia (P/p)	55 (28/27)	—	—	—	140 (69/71)	74 (35/39)	209 (101/108)	—	—	—	478 (233/245)
Elevation in Ca × P product levels (P/p)	—	—	—	—	140 (69/71)	74 (35/39)	209 (101/108)	—	—	—	423 (205/218)
Reduction in proteinuria	55 (28/27)	22 (15/7)	—	—	—	—	—	—	—	272 (184/88)	349 (227/122)

P: Paricalcitol group; p: placebo group.

**Table 2:** The quality of the nine studies.

Study year	Research method	Randomization	Blinding	Allocation concealment	Withdrawal and loss	Intention to treat analysis	Baseline demographic characteristics	Jadad score	Quality
Fishbane et al. [4], 2009	Prospective, randomized, placebo-controlled, double-blinded trial	Computer generated	Double- blinded	Adequate	Description	Used	Similar	5	A

Alborzi et al.[5], 2008	A randomized, double-blinded pilot trial	Computer generated	Double- blinded	Adequate	Description	Used	Similar	5	A

Agarwal et al.[6], 2005	Three, randomized, placebo-controlled, double-blinded trials	—	Double- blinded	Unclear	—	Unclear	Similar	2	B

Martin et al.[7], 1998	Randomized, placebo-controlled, double-blinded multi-investigator study	—	Double- blinded	Unclear	No	—	Similar	3	B

Abboud et al.[8], 2006	Three, prospective, randomized, placebo-controlled, double-blinded multicenter studies	—	Double- blinded	Unclear	—	Unclear	Similar	2	B

Coyne et al.[9], 2006	Three, randomized, placebo-controlled trials	Computer generated	Double- blinded	Adequate	—	Unclear	Similar	4	B

Greenbaum et al.[10], 2007	Randomized, placebo-controlled, double-blinded trial	—	Double- blinded	Unclear	Description	Unclear	Similar	3	B

Ross et al.[11], 2007	Randomized, placebo-controlled, double-blinded trial	Computer generated	Double- blinded	Adequate	—	Unclear	Similar	3	B

De Zeeuw et al. [12], 2010	A multicenter randomized placebo-controlled, double-blinded clinical trial	Computer generated	Double- blinded	Adequate	Description	Used	Similar	5	A

**Table 3:** Meta-analysis.

Figures			Heterozygosity test					
	Paricalcitol	Placebo	*χ* ^2^	*P*	Pooled RR	95% CI	*Z*	P
Figure 1	369	351	3.28	0.77	6.97	5.27–9.23	13.57	<0.00001
Figure 2	221	247	420.01	<0.00001	—	—	—	—
Figure 3	227	122	3.72	0.16	1.57	1.20–2.04	3.29	0.001
Figure 4	100	99	0.48	0.49	1.04	0.81–1.33	0.32	0.75
Figure 5	495	380	0.64	0.96	2.91	0.86–9.90	1.71	0.09
Figure 6	233	245	0.6	0.9	0.94	0.56–1.58	0.22	0.82
Figure 7	205	218	0.3	0.86	1.97	1.06–3.67	2.15	0.03

**Table 4:** Comparison of the mean change in eGFR from baseline to final visit (ΔeGFR).

Studies	ΔeGFR (mL/min/1.73 m^2^) ± SD		
	Paricalcitol group	Placebo group	*P* value
Agarwal et al. [6]	−2.5 ± 0.54	−3.0 ± 0.53	*P* = 0.51
Abboud et al. [8] (TIW)	−2.9 ± 0.61	−1.3 ± 0.60	*P* = 0.066
Abboud et al. [8] (QD)	−1.6 ± 1.01	−2.0 ± 0.88	*P* = 0.78
Coyne et al. [9]	−2.52 ± 0.526	−1.57 ± 0.494	*P* = 0.187

**Table 5:** Change in calcium × phosphorus product levels.

Reference	Paricalcitol group	Placebo group	*P* value
	*n*/*N*	*n*/*N*	
Abboud et al. [8] (QD)	5/35	2/39	0.245
Abboud et al. [8] (TIW)	8/69	5/71	0.396
Coyne et al. [9]	13/101	7/108	0.161
